# Label-free candidate dOCM biomarkers associated with blastocyst quality using total cell count as a proxy

**DOI:** 10.1364/BOE.599635

**Published:** 2026-06-03

**Authors:** Yunqin Zhao, Saanjali Majmundar, Jacob Stoebner, Audrey K. Bowden

**Affiliations:** 1 Vanderbilt University, Vanderbilt Biophotonics Center, Department of Biomedical Engineering, Nashville, TN 37232, USA; 2 Vanderbilt University, Vanderbilt Institute for Surgery and Engineering, Nashville, TN 37232, USA; 3 Vanderbilt University, Department of Electrical and Computer Engineering, Nashville, TN 37232, USA

## Abstract

Blastocyst grading during *in vitro* fertilization relies on subjective morphology, motivating quantitative, label-free biomarkers measurable in blastocysts. We apply dynamic optical coherence microscopy (dOCM) to murine blastocysts and quantify intracellular fluctuation spectra using full- and band-resolved metrics (mean frequency, RMS bandwidth, motility amplitude, and variance fraction). Using cell counting as a benchmark, full-band metrics show no significant differences between low- and high-cell-count cohorts. In contrast, band-resolved analysis reveals higher mid-band mean frequency and increased mid-band RMS bandwidth in higher-cell-count embryos, accompanied by a significant decrease in low-frequency variance fraction. These exploratory results suggest frequency-specific dOCM signatures as candidate biomarkers for blastocyst quality.

## 1. Introduction

One hundred eighty million couples worldwide suffer from infertility [1]. In the US, more than one in eight women aged 15 to 44 have trouble carrying a pregnancy to term [2]. While access to *in vitro* fertilization (IVF) is available, the live birth rate for IVF persists at a low 37.5%, according to the Centers for Disease Control and Prevention report in 2022. Since embryo quality correlates with the chance of pregnancy [3–5] and live birth [6,7], choosing the embryo of the best quality for transfer is crucial.

During IVF procedures, embryologists determine embryo quality mainly by assessing morphological appearance using a brightfield microscope. To optimize this selection process, clinics increasingly extend culture to the blastocyst stage (days 5–6), which enables embryonic self-selection and better synchronizes embryo transfer with endometrial receptivity. Because this approach yields higher implantation and live birth rates compared to cleavage-stage transfers, assessment is now predominantly performed at this later stage. During standard blastocyst grading, an embryologist evaluates embryos using the Gardner grading system [8], which comprises an expansion grade (1–6) based on blastocoel expansion (i.e., size) and hatching status and separate grades (A-C) for the inner cell mass (ICM) and trophectoderm (TE), both based on visual assessment of apparent cell abundance and compactness. Although this grading method was shown to predict the pregnancy rate [9,10], the process is subjective and can, therefore, suffer from inter-observer and intra-observer variability [11], which limits the grading precision.

To address these limitations, some clinics have adopted a time-lapse incubator microscope capable of providing objective information about dynamic processes in the embryo. By imaging the embryo every few minutes or hours via brightfield or darkfield microscopy [12], these microscopes capture embryo dynamics; several models can even capture 3D information about the embryo’s morphology [13]. The timing information these systems provide enables the calculation of morphokinetic biomarkers (e.g., time to two-cell stage, time to full blastocyst) that can be used to characterize embryo grade objectively. As such, these timing-based metrics provide an approach to embryo assessment that is distinct from direct evaluation of blastocyst morphology. Although these measurements reduce inter- and intra-observer variability, randomized clinical studies have not consistently demonstrated that time-lapse–derived morphokinetic parameters improve live birth rates compared to standard morphological assessment [14,15]. Moreover, time-lapse systems remain limited to tracking macroscopic morphological events and division timing and do not provide information about subcellular functional dynamics such as intracellular transport, metabolic activity, or organelle motion. Thus, while time-lapse imaging enhances measurement objectivity at the morphological level, it does not directly measure intracellular functional dynamics that may reflect embryo quality.

Some researchers have introduced more sophisticated imaging methods, such as hyperspectral imaging [16] and fluorescence lifetime imaging [17], in an effort to provide subcellular morphological insights for improving embryo grading in a label-free manner. Label-free imaging is particularly important in IVF because labeling and post-fixation analysis are incompatible with subsequent embryo transfer and therefore limits clinical applicability. Optical coherence microscopy (OCM), an adaptation of optical coherence tomography (OCT) capable of visualizing mitochondrial distribution [18] and spindle structure [19,20], has also been used to visualize pre-implantation embryos in 3D without labeling. A recent study showed that OCM enables label-free counting of the number of cells in embryonic day-three mouse embryos (i.e., compacted embryos) and that the number of cells in compacted mouse embryos correlates with blastocyst rate, hatching capability, and number of cells in the blastocyst [21]. This result suggests that early-stage cell counts can inform the embryo quality at blastocyst stage; however, this association is not determinative. Developmental trajectories can diverge between the cleavage/compaction stage and the blastocyst stage, so a high-quality day-three embryo does not necessarily yield a high-quality day-five embryo (i.e., a blastocyst). Thus, day-three cell count remains an indirect and less accurate proxy for blastocyst-stage status and does not provide a quantitative readout obtainable directly in blastocysts. Because embryo selection and transfer are commonly performed at the blastocyst stage, there is a clinical need for quantitative, non-invasive assessment tools applicable at the blastocyst stage. Unfortunately, extending the OCM cell counting to blastocyst stage embryos presents unique challenges. Unlike the simple geometry of cleavage-stage embryos, the blastocyst consists of hundreds of tightly packed cells. In this dense structure, the refractive index contrast between cells is low, and signal attenuation limits the ability of standard OCM to resolve individual cell boundaries for counting. Despite these challenges, structural OCT-based analysis of blastocysts has been systematically attempted. A systematic evaluation of 22 structural parameters — including ICM, TE, and zona pellucida (ZP) thickness and volume, as well as blastocoel geometry — derived from 3D OCT images of bovine blastocysts found no significant association with pregnancy outcome after embryo transfer, and only modest discrimination of in vitro hatchability [22–24], suggesting that static structural morphometry alone has limited discriminative power for blastocyst quality assessment. A recent pilot study successfully applied 3D label-free imaging using OCM to human blastocysts [25]; however, likely due to these structural complexities, the analysis remained tethered to subjective morphological grading rather than extracting quantitative biomarkers. Consequently, while biomarkers exist for earlier stages, a quantitative OCM biomarker for blastocyst quality remains undefined.

To overcome the limitations of structural imaging, dynamic OCM (dOCM) — an extension of OCM that tracks the fluctuations of particle back-scattering related to intracellular motions, metabolic processes, and other cellular activities [26,27] — enables quantification of functional intracellular dynamics that are not accessible through the static intensity imaging of conventional OCM. The capability of dOCM to quantify intracellular dynamics shows significant potential for blastocyst quality assessment. While a previous study demonstrated the use of dOCM for cytoplasmic motion tracking in early-stage mouse embryos [28], this approach has never been leveraged for quantitative blastocyst quality assessment. In this study, we use total cell count at the blastocyst stage — measured with a nuclear counting assay — as a quantitative proxy for blastocyst quality, as it is a well-established proxy for blastocyst developmental potential and implantation success [29–31]. We hypothesize that dOCM-derived intracellular activity metrics can serve as biomarkers that correlate with this cellular benchmark, thereby providing a non-invasive, functional surrogate for total cell count and enabling quantitative assessment without the need for destructive labeling or post-fixation analysis.

We present the first application of dOCM to derive quantitative biomarkers for blastocyst quality assessment based on intracellular dynamic signatures rather than static morphological features. To enable this, we developed a custom OCM sub-system featuring a modular sample arm integrated into a commercial inverted microscope platform commonly used in IVF laboratories, permitting dOCM imaging within a standard embryology workflow. We imaged blastocyst-stage mouse embryos and objectively assessed their quality using total cell count as a quantitative benchmark. Through power spectrum analysis (PSA) and spectral decomposition, we evaluated intracellular fluctuation dynamics across various frequency bands.

The goal of this work is to establish the potential of dOCM to provide a quantitative, functional readout of embryo viability that is inaccessible through standard morphological grading. By leveraging dynamic, motility-based biomarkers, our approach overcomes the inherent limitations of standard structural OCM imaging in dense cellular environments. Rather than relying solely on static morphology, these sub-band metrics capture the complex metabolic and transport dynamics that are fundamentally indicative of blastocyst quality. We propose that the identification of spectral biomarkers can facilitate a more accurate and quantitative grading system. Thus, translation of dOCM to clinics may improve chance of pregnancy and live birth.

## 2. Methods

### 2.1. Optical coherence microscope

We developed an OCM system integrated into a commercial microscope (DMI8, Leica) equipped with a stage-top incubator (ECU-HOC-IV, In Vivo Scientific) to maintain temperature and CO_2_ levels that minimize the disturbance of the embryos during imaging.

**Fig. 1.**
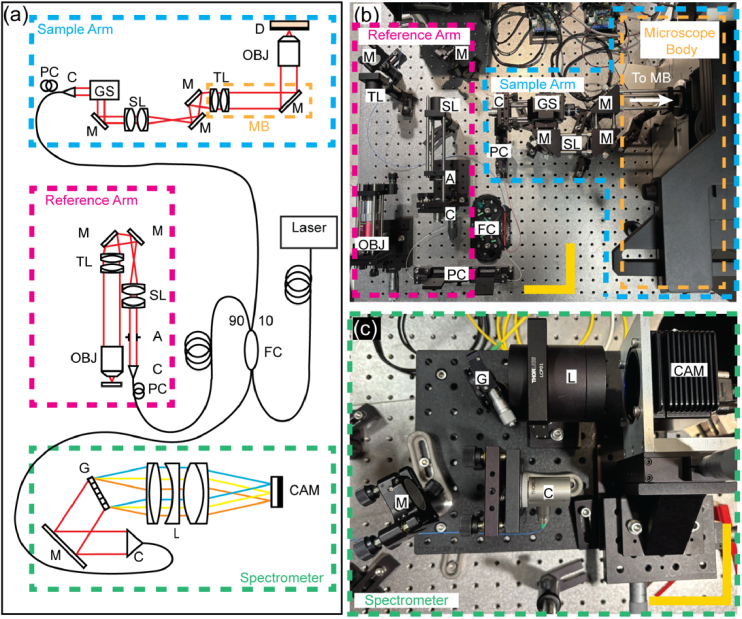
Optical coherence microscopy (OCM) system configuration. (a) Schematic of the spectral domain OCM system microscope (fiber coupler, FC; polarization controller, PC; collimator, C; galvanometer scanner, GS; mirror, M; scan lens, SL; tube lens, TL; objective lens, OBJ; grating, G; lens, L; camera, CAM; microscope body, MB; dish, D). The schematic is not drawn to scale. The reference arm is used solely for system characterization measurements (resolution, sensitivity, fall-off and stability) and is not used during embryo imaging, during which the reference arm aperture is closed for self-interference acquisition. (b) Photograph of the OCM integrated with a commercial microscope. The tube lens and objective lens of the OCM sample arm are not shown in the picture. (c) Photograph of the home-built spectrometer. (scale bar: 10 cm)

The OCM system utilized a fiber-based spectral-domain optical coherence tomography (SDOCM) [32] configuration and included a fiber coupler, a laser, a reference arm, a sample arm, and a spectrometer. Figure 1 shows the OCM system configuration. We employed a supercontinuum light source (SuperK Extreme EXW-4, NKT) with a longpass filter (FEL0700, Thorlabs) and a shortpass filter (FES0900, Thorlabs) to yield a spectrum centered at 800 nm with a 200 nm bandwidth. The light was then coupled via fiber-delivery system (SuperK CONNECT, NKT) into a single-mode fiber that was connected to a 2 × 2 90:10 fiber coupler (TW850R2A2, Thorlabs). Polarization controllers (FPC030, Thorlabs) were added in both the reference arm and sample arm to optimize the interference signal intensity at the detector, as is standard practice in fiber-based OCM systems. The tap output port (i.e., 10% output port) of the fiber coupler was connected to the sample arm so that 90% of light from the sample could be coupled to the spectrometer to improve the signal-to-noise ratio (SNR). In the sample arm, light launched by the fiber was first collimated by a collimator package (RC02APC-P01, Thorlabs) and made incident on a galvanometer scanner (GVS002, Thorlabs) that was controlled by a data acquisition device (DAQ, NI USB-6211, National Instrument) to perform X-Y scanning. A scanning lens comprising two achromatic lenses (AC254-200-B, Thorlabs) and a tube lens comprising two achromatic lenses (AC254-400-B, Thorlabs) were paired to magnify the beam diameter and to relay the light to the objective (378-824-5, Mitutoyo; 20X, NA 0.40) for imaging. The signal output port (i.e., 90% output port) was connected to the reference arm, which hosted the same optical configuration as that in the sample arm, excluding the galvanometer scanner. Additionally, an iris diaphragm was added after the collimator to adjust the light power in the reference arm. Then, reflections from the sample and reference arms interfered in the fiber coupler and were delivered to a home-built spectrometer. In the spectrometer, the interfered light was collimated by a collimator package (RC08APC-P01, Thorlabs) and reflected by a folding mirror (PF10-03-P01, Thorlabs) to a holographic transmission grating (GP1012 L, Thorlabs) to separate different wavelengths of light. A three-lens group (two AC508-075-B, LC1093-B, Thorlabs) then focused the light to a line scan camera (Sprint spl2048km, Basler) that was connected to a frame grabber (Xcelera-CL LX1, Teledyne) for control and acquisition. To control the OCM system, we utilized an open-source SDOCM control software (OCTSharp) [33] for data collection and real-time display. For image reconstruction, we applied a standard OCM data processing pipeline comprising spectral reshaping, linearization, and Fourier transform to reconstruct the images. We validated the imaging resolution and system sensitivity by using a USAF resolution test target and by measuring the point-spread function response of the system to a mirror, respectively. To characterize system stability, we performed a dedicated measurement using a mirror target placed at a slight angle relative to the beam axis. This configuration caused the mirror reflection to appear as a diagonal line across the B-scan, enabling tracking of the mirror peak position at each A-scan independently. We acquired 100 repeated B-scans at the same location, matching the acquisition protocol used for embryo dOCM imaging (see Embryo dOCM Imaging section). The mirror peak location was identified at each A-scan for each frame, yielding a 256 × 100 matrix of peak positions. To isolate the mirror peak signal from the noise background, a window of ±5 pixels centered on the peak location was extracted at each A-scan, with all remaining pixels set to zero. Cross-correlation analysis was then performed between each frame and the first frame to quantify frame-to-frame lateral and axial displacement.

### 2.2. Embryo culture

Cryopreserved single-cell mouse embryos (B6C3F1 × B6D2F1, EmbryoTech) were employed in this study. We followed a standard culture protocol for mouse embryos [34]. On day 0, we prepared an IVF dish (150255, Thermo Fisher) containing a droplet of 80 μL of culture media (MR-101-D, Sigma) covered with 4 ml of culture oil (90189, Irvine Scientific) and kept the dish in an incubator (3110, Thermo Fisher, US) at 37 °C under 6% CO_2_ overnight for pre-equilibration. The 6% CO_2_ level was based on our laboratory's existing mouse embryo culture conditions.

On day 1, a straw of embryos was exposed to room temperature for two minutes and immersed in a 37 °C water bath for one minute. Then the straw was moved from the water bath and wiped dry with a tissue. We cut the straw at the plug and seal and pushed all 10 embryos into a droplet of 250 μL of embryo culture media that was pre-warmed in an IVF dish. After that, we immediately rinsed the embryos twice in two droplets of 250 μL of pre-warmed culture media in two separate IVF dishes set aside for rinsing. The embryos were left in the second rinsing droplet and incubated for 10 minutes in the incubator at 37 °C under 6% CO_2_ to rehydrate. Warmed embryos were then loaded into the pre-equilibrated culture dish prepared on day 0. The embryos were cultured in an incubator at 37 °C under 6% CO_2_ for four days until the blastocyst stage. All embryo handling was performed under a stereomicroscope (SM-2BYY, AmScope) on a warm plate at 37 °C (TCS-100, AmScope) using a micropipette (7-72-2170, Cooper Surgical). In this study, we conducted four repetitions, and the number of embryos cultured in each repetition is summarized in Table 1. As expected, not all embryos developed into blastocysts. Only embryos that developed to the blastocyst stage (76%) were included for dOCM imaging.

**Table 1.** Summary of embryo culture and imaging yield per experimental repetition (only embryos that developed to the blastocyst stage were imaged).

Repetition	Cultured embryos	Imaged embryos
1	12	9
2	11	11
3	12	8
4	10	6
Total	45	34

### 2.3. Embryo dOCM imaging

To facilitate identification of each embryo during both dOCM and the subsequent nuclear counting assay (a fluorescence-based measurement), we placed individual embryos in separate droplets of culture media. On day 3, we loaded one to three labeling droplets (6 µl each) on the plastic area of glass-bottom dishes (P35G-1.0-20-C, MatTek), with the number of droplets identifying each dish (first through third). We then placed four 20 µl droplets of culture media on the glass area of the dishes to house embryos and covered them with 3 ml of culture oil. The dishes were incubated overnight at 37 °C under 6% CO2 for pre-equilibration. After four days of embryo culture, we collected embryos from the IVF dish and placed each embryo individually into separate 20 µl droplets of culture media in the prepared glass-bottom dishes. We excluded any embryos that had not developed to the blastocyst stage during this process. We then transported the dishes to the stage-top incubator at 37 °C under 6% CO_2_ for imaging. The number of embryos that was imaged is summarized in Table 1.

For dOCM imaging, we performed a 256 × 256-point scan covering an area of 151 µm × 151 µm for each embryo. The A-scan rate was configured to 46.5 kHz with 20 µs exposure time. The fast-axis galvanometer scanner flyback period was set to 150 points, resulting in an equivalent B-scan rate (i.e., sampling rate, $F_{s}$ ) of 114.5 Hz and a corresponding Nyquist frequency ( $F_{s}/2$ ) of 57.25 Hz. We collected 100 repetitive scans at each B-scan location to capture dynamic information, with each A-scan recorded with 2048 pixels in the raw interferogram, yielding 1024 depth pixels after Fourier transform. This volumetric acquisition — spanning all 256 y-positions — yields a 4D dataset (256 × 256 × 1024 × 100) per embryo and ensures that dOCM metrics are sampled across the full extent of the embryo rather than at a single imaging plane. The laser power was set to provide 1 mW after the objective, yielding a power density of approximately 9.8 kW/cm^2^. All embryo dOCM imaging was performed in self-interference mode, in which the aperture in the reference arm was closed to avoid saturation artifacts from the strong interference between the coverslip reflections and the reference arm signal. The reference arm was retained in the system solely for system characterization measurements, including resolution, sensitivity, fall-off and stability measurements reported in the OCM Characterization section. Both layers of the coverslip reflect light and interfere with the embryo reflections, resulting in two identical embryo images at different depths in each B-scan, known as multiple reference OCT (MR-OCT) [35]. The two images were generated simultaneously within a single interferometric (i.e., A-scan) acquisition and therefore corresponded to the same temporal state of the embryo.

### 2.4. Embryo staining and cell counting

We counted the number of cells in each embryo using a nuclear staining assay. Note that while this fluorescence assay required labeling, the labeling was done merely to serve as a ground truth to correlate with dOCM parameters. To minimize post-imaging culture duration and ensure all embryos were fixed at a consistent developmental stage, we prepared three components prior to dOCM imaging: rinsing buffer, staining solution, and confocal imaging dishes. For the rinsing buffer, we diluted 1% polyvinylpyrrolidone (PVP) in phosphate-buffered saline (PBS) buffer (DCP-PVPPBS1X, Diagnocine) with PBS (10010049, Thermo Scientific) to create a 0.1% PVP-PBS solution. This PVP prevents embryos from sticking to the plastic dish during transfers between droplets. For the staining solution, we diluted a DNA-binding dye (Hoechst 333342, 10 mg/ml, H3570, Thermo Scientific) in PVP-PBS to a working concentration of 5 mg/ml. Finally, we prepared confocal fluorescence imaging dishes using the same method as for dOCM imaging dishes, except we used PVP-PBS instead of culture media for the 20 µl droplets.

After dOCM imaging, we transferred the dishes from the incubator to a biosafety cabinet. We individually rinsed each embryo twice in 50 µl droplets of PVP-PBS and fixed them in 4% paraformaldehyde (PFA, J19943.K2, Thermo Scientific) for 30 minutes. Following fixation, the embryos were rinsed twice more in 50 µl droplets of PVP-PBS and stained in 50 µl droplets of dye solution for 30 minutes. Finally, we rinsed the embryos twice in 50 µl droplets of PVP-PBS and placed each embryo into a PVP-PBS droplet in the prepared imaging dishes for subsequent confocal imaging.

We transported the dish to a confocal microscope (AX R, Nikon) for fluorescence imaging using a 405-nm laser. We performed volumetric scanning on each embryo individually. The confocal images were then loaded into Elements software (Nikon), where we used General Analysis 3 to identify and count the bright spots, each corresponding to one embryonic cell nucleus. In the software, we set the expected object diameter to 10 µm and adjusted the contrast to ensure all visible bright spots were included in the count.

### 2.5. OCM image processing and manual segmentation

**Fig. 2.**
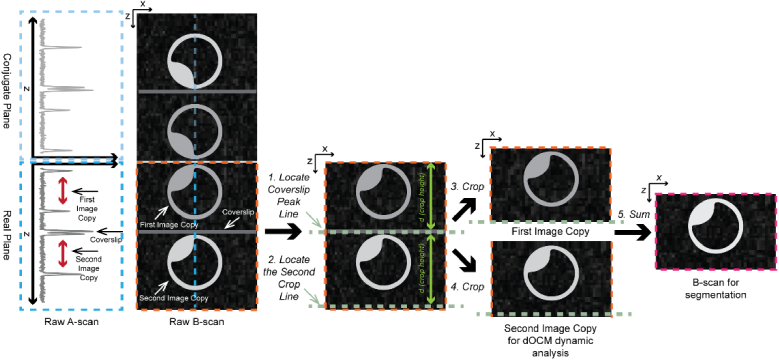
Schematic of B-scan processing workflow. The raw B-scan contains two copies of the embryo image in the real plane, separated by the coverslip self-interference peak, along with a conjugate plane that is excluded from processing entirely. An A-scan through the center of the B-scan, showing the corresponding peak locations, is shown on the left. The raw B-scan is first segmented at the location of this peak to delineate the two image copies. The vertical dimension, *d*, determined from the first image copy, defines the cropping boundary for the second image copy. The second image copy is used for all dOCM dynamic analysis. It is formed by interference with the glass-air interface, which has higher reflectivity than the glass-medium interface that forms the first image copy, resulting in higher SNR despite the greater imaging depth. The two cropped copies are spatially aligned and summed to produce the compounded B-scan, which is used solely for generating the averaged intensity map for manual segmentation.

We loaded the raw interferogram data into MATLAB (MathWorks) and applied standard OCT processing to reconstruct B-scans, which we term “raw B-scans.” Each raw B-scan contains both a conjugate plane and a real plane; only the real-plane images were used for further processing. To enhance SNR, we leveraged information from the two copies of the embryo image present in each raw B-scan to generate a compounded B-scan for segmentation (Fig. 2). This process involved first identifying the self-interference peak of the coverslip in the first A-scan of each raw B-scan. We then split the raw B-scan by identifying the peak location of the coverslip signal. Using the height of the first region (*d*) as a reference, we determined a crop line to apply to the deeper embryo image to make the images the same height. Finally, we aligned and summed the two copies of the embryo images to produce a compounded B-scan with improved signal. The compounded B-scan generation process was applied to all raw B-scans. We then averaged 100 repeated compounded B-scans to generate a higher-SNR intensity image for each embryo.

For dOCM dynamic analysis, we extracted the second image copy from each raw B-scan to avoid ambiguity introduced by compounding two image copies with unequal SNR contributions. In our self-interference MR-OCT geometry, the second image copy is formed by interference between the glass-air interface (reflectivity ∼4.1% based on Fresnel equations) and the embryo signal, while the first image copy is formed by interference between the glass-medium interface (reflectivity ∼0.41% based on Fresnel equations) and the embryo signal. Because the second copy is positioned deeper, it incurs a fall-off penalty of approximately 0.86 dB, estimated based on the 6-dB roll-off depth at an image depth difference of ∼226.5 µm calculated as the optical path distance of the No. 1 coverslip used in this study (ideal coverslip thickness × refractive index at 800 nm = 150 µm × 1.51 = 226.5 µm). Nevertheless, the intrinsic reference reflectivity advantage of 10.25 dB results in a net SNR advantage of approximately 9.41 dB for the second copy over the first, making it the higher-SNR choice for dOCM dynamic analysis.

**Fig. 3.**
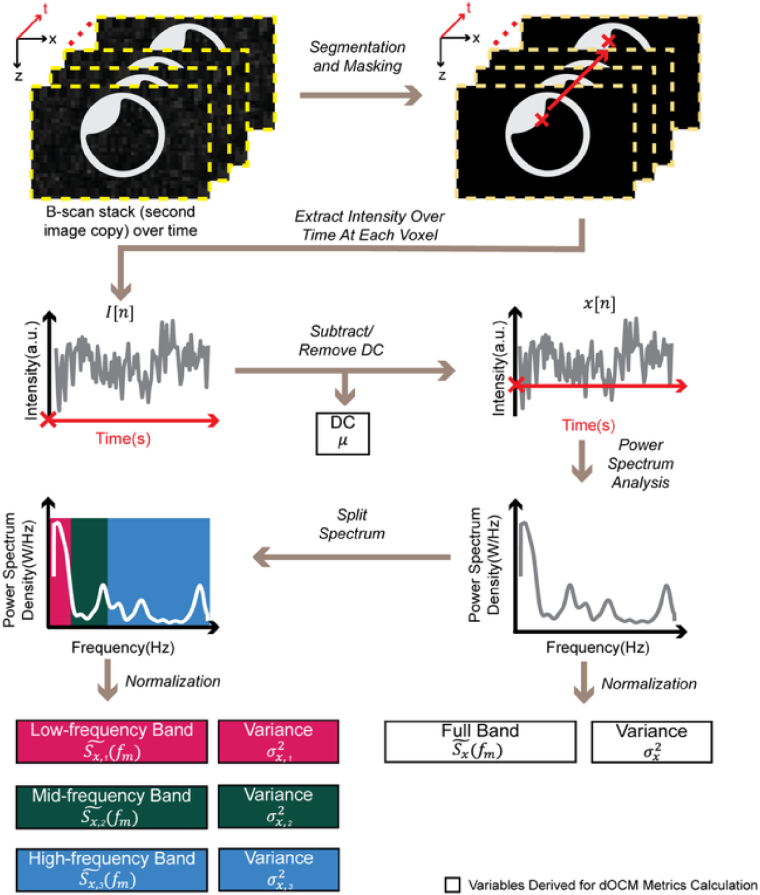
Derivation of variables used for dynamic optical coherence microscopy (dOCM) metrics calculations. Repeated second-copy B-scans acquired over time at each y-position are first segmented and masked to isolate the blastocyst from the background. For each voxel within the segmented region, the temporal intensity signal is extracted across repeated scans, and the static DC component is removed to obtain the zero-mean fluctuation signal. The power spectral density (PSD) is then computed. The full-band spectrum is normalized, and variance is calculated via Parseval’s theorem. The spectrum is subsequently divided into predefined low-, mid-, and high-frequency sub-bands, each independently normalized for band-specific variance estimation. The DC component normalized full-band spectrum, sub-band spectra, and associated variance terms are then used in Eqs. (2)–(12) for dOCM metric calculations.

To isolate the cellular region including the TE and ICM and to define a binary region of interest (ROI) for analysis, we performed semi-automated segmentation of the 3D OCM intensity volumes (Fig. 3). Image preprocessing and post-processing were conducted in MATLAB (MathWorks), while volumetric segmentation was performed in 3D Slicer [36,37]. First, pixel intensities were normalized to the range (0, 1) using min-max scaling, and adaptive histogram equalization was applied to each axial slice to enhance contrast. The processed volumes were then exported to 3D Slicer for segmentation. In 3D Slicer, the blastocoel (i.e., cavity) region was segmented using a manual low-intensity threshold followed by slice-by-slice refinement. The Surface Wrap Solidify [38] extension was applied to fill internal holes. The full blastocyst was segmented separately using a high-intensity threshold refined in the same manner as the blastocoel. The blastocoel mask was then subtracted from the full embryo segmentation, yielding two non-overlapping segmentations corresponding to the blastocoel and cellular region. A Joint Smoothing (smoothing factor = 1) was applied to both segmentations.

### 2.6. dOCM metric calculation

To characterize the time-dependent fluctuations of the OCT signal, quantitative spectral analysis was performed on time-intensity profiles extracted from repetitive B-scans, following the previously established protocols [28,39–42]. The analysis pipeline was implemented in MATLAB (MathWorks). To minimize computational overhead, only voxels within the ROI were processed. Figure 3 summarizes the processing steps applied to extract the variables used for dOCM metric calculation at each voxel. These variables were then used to compute the dOCM metrics.

#### 2.6.1. Full-band power spectral density (PSD) analysis

For every voxel, the temporal intensity signal I[n](n=0,1,…,N−1,withN=100) was extracted from the sequence of B-scans. To isolate dynamic fluctuations from the static background, we first calculated the temporal mean (DC component), μ:

(1) $$\mu =\frac{1}{N}\sum_{n=0}^{N-1}I[n].$$

The zero-mean fluctuation signal was then defined as x[n]=I[n]−μ . Power spectral densities (PSDs) were estimated from x[n] using Welch’s method; this approach minimizes spectral leakage through Hanning windowing and lowers estimator variance via segment averaging. This yielded a one-sided PSD, $S_{x}(f)$ , sampled at discrete frequency points $f_{m}\;(m=1,\;\ldots ,\;M,\;\text{with}\;M=1024)$ . To compare spectral shape information (i.e., dynamic information) independently of scattering intensity, each PSD was L1-normalized to unit area using trapezoidal numerical integration over frequency:

(2) $$\tilde{S_{x}}(f_{m})=\frac{S_{x}(f_{m})}{\Delta f\left[\frac{1}{2}S_{x}(f_{1})+\sum_{k=2}^{M-1}S_{x}(f_{k})+\frac{1}{2}S_{x}(f_{m})\right]},$$

where the $\Delta f=\;F_{s}/(2\times M)$ is the frequency resolution.

From this normalized distribution, we extracted the mean frequency ( $f_{mean}$ ) and RMS bandwidth ( $f_{RMS}$ ) to quantify the characteristic speed and frequency spread of the underlying motion, respectively:

(3) $$f_{mean}=\sum_{m=1}^{M}f_{m}\tilde{S_{x}}(f_{m})\Delta f,$$

(4) $$f_{RMS}=\sqrt{\sum_{m=1}^{M}{\left(f_{m}-f_{mean}\right)}^{2}\tilde{S_{x}}(f_{m})\Delta f}.$$

To quantify the total fluctuation magnitude while minimizing computational overhead, we utilized Parseval’s theorem, which equates the total fluctuation variance ( $\sigma_{x}^{2}$ ) to the integrated spectral power. By calculating the variance directly in the frequency domain, we eliminated the need for redundant time-domain computations, providing a measure of the total dynamic energy at each voxel:

(5) $$\sigma_{x}^{2}=\int_{0}^{F_{s}/2}S_{x}(f)df\approx \sum_{m=1}^{M}S_{x}(f_{m})\Delta f.$$

Finally, the motility amplitude ( M ) was calculated to remove the impact of scattering intensity and represent the relative strength of the dynamic component compared to the static scattering background ( μ ):

(6) $$M=\frac{\sqrt{\sigma_{x}^{2}}}{\mu }.$$

#### 2.6.2. Sub-band PSD analysis

To distinguish between different regimes of intracellular motion, the frequency spectrum was segmented following dynamic OCT and tissue dynamics spectroscopy (TDS) frameworks established in 3D tissue models [43–45]. Prior studies have demonstrated that fluctuation spectra exhibit frequency-dependent responses to metabolic and cytoskeletal perturbations, supporting the use of band-resolved analysis to probe distinct motility timescales. Based on these studies, slower motions (0.1–1.0 Hz) are commonly interpreted as reflecting larger-scale membrane and structural dynamics [43]. Mid-frequency motions (1–5 Hz) have been shown to be sensitive to metabolic inhibition, consistent with their association with ATP-dependent intracellular transport processes, including motor-driven organelle and vesicle trafficking [43,44]. Higher-frequency motions (>5 Hz) are likely associated with more rapid, small-scale subcellular fluctuations, including stochastic vesicle motion and diffusion-like processes [39,45]. Guided by this framework, we adopted this spectral decomposition to characterize the murine embryo. Accordingly, the spectrum was divided into three distinct sub-bands: low-frequency (0.1–1.0 Hz), mid-frequency (1.0–5.0 Hz), and high-frequency (5.0–57.25 Hz). Importantly, these frequency bands do not directly correspond to specific cellular processes; moreover, their biological interpretation has not been experimentally validated in murine blastocysts. Rather, the sub-band decomposition provides a principled way to separate intracellular motion into distinct time-scale regimes. Accordingly, we interpret these differences as frequency-dependent signatures of intracellular activity and assess their association with cell number as a proxy of blastocyst quality.

For each sub-band *i*, the PSD was first self-normalized within its respective frequency range $\left[f_{start,\;i},\;f_{end,\;i}\right]$ to isolate localized spectral characteristics, independently of signal magnitude, using trapezoidal numerical integration over frequency. Let the sub-band *i* contain frequency indices $k=\;k_{i,1},\;\cdots ,\;k_{i,N_{i}}$ , then:

(7) $$\tilde{S_{x}}(f_{m})=\frac{S_{x}(f_{m})}{\Delta f\left[\frac{1}{2}S_{x}(f_{k_{i,1}})+\sum_{k=k_{i,1}\;}^{M-1}S_{x}(f_{k})+\frac{1}{2}S_{x}(f_{k_{i,N_{i}}})\right]}\;\text{for}\;m\in band_{i}$$

From these self-normalized distributions, we calculated the sub-band mean frequency ( $f_{mean,\;i}$ ) and sub-band RMS bandwidth ( $f_{RMS,\;\;i}$ ) to describe the localized dynamics:

(8) $$f_{mean,\;i}=\sum_{m\in \text{band}\;i}f_{m}\tilde{S_{x,\;i}}(f_{m})\Delta f,$$

(9) $$f_{RMS,\;i}=\sqrt{\sum_{m\in \text{band}\;i}{\left(f_{m}-f_{mean,\;i}\right)}^{2}\tilde{S_{x,\;i}}(f_{m})\Delta f}.$$

To quantify the total dynamic energy within each regime, the sub-band variance ( $\sigma_{x,\;i}^{2}$ ) was derived using Parseval’s theorem:

(10) $$\sigma_{x,\;i}^{2}=\int_{f_{start,\;i}}^{f_{end,\;i}}S_{x}(f)df\approx \sum_{m\in \text{band}\;i}S_{x}(f_{m})\Delta f.$$

The sub-band motility amplitude ( $M_{i}$ ) was defined as the ratio of the sub-band fluctuation magnitude to the DC intensity ( μ ):

(11) $$M_{i}=\frac{\sqrt{\sigma_{x,\;i}^{2}}}{\mu }.$$

Additionally, the relative contribution of each regime to the total dynamic signal was determined by the variance ratio (or motility fraction), $R_{i}$ :

(12) $$R_{i}=\frac{\sigma_{x,\;i}^{2}}{\sigma_{x}^{2}}.$$

The variance ratio $R_{i}$ was calculated to quantify the relative distribution of dynamic energy across different physiological timescales. To mitigate the confounding effects of scattering variations caused by the sample or the imaging system, the analysis was restricted to scattering-independent dOCM metrics: mean frequency, RMS bandwidth, motility amplitude, and variance ratio.

#### 2.6.3. Global ROI averaging

To derive a global quantitative index for each sample, the pixel-wise dOCM metrics were then spatially averaged across the entire segmented volume. These global average values served as the representative dOCM markers for the following statistical analysis.

#### 2.6.4. Statistical analysis

Statistical evaluation and graphing were performed using OriginPro (OriginLab Corporation, Northampton, MA). The normality of the data distribution was assessed using the Shapiro-Wilk test, and due to the non-normal distribution of the dOCM metrics, a non-parametric test was employed. Note that total cell count was used as the proxy for blastocyst quality for the following correlation and group-comparison analyses.

To validate the utility of dOCM as a viability metric, Spearman’s rank correlation coefficients (r_s_) were first calculated to assess the relationship between the cell count and the dOCM spectral metrics, including mean frequency, RMS frequency bandwidth, motility amplitude, and variance fraction. This correlation analysis evaluates whether each metric changes monotonically with cell count. To account for multiple comparisons across the 15 correlation tests, the Benjamini-Hochberg False Discovery Rate (BH-FDR) correction [46] was applied, with a significance threshold of FDR < 0.05.

To complement the correlation analysis, we also evaluated whether dOCM metrics could distinguish discrete developmental phenotypes. Because there is no standardized or universally accepted cell number threshold to categorize murine blastocysts into discrete high- or low-quality cohorts, the cohort was stratified into two independent groups — high- and low-cell count-groups — based on the median cell count (n = 58). Our goal here was to characterize distinct developmental phenotypes without introducing the selection bias associated with arbitrary cutoffs. Whereas correlation probes continuous trends across the full range, the stratified analysis tests for distributional differences between cohorts and may be sensitive to threshold-like shifts. Comparisons of the spectral metrics between these two independent groups were performed using the two-tailed Mann-Whitney U test. To account for multiple comparisons across the 15 group-comparison tests, the BH-FDR correction was applied, with a significance threshold of FDR < 0.05.

#### 2.6.5. Intra-embryo spatial heterogeneity of dOCM metrics

To quantify spatial heterogeneity within individual embryos, we additionally computed the coefficient of variation (CV = Standard Deviation/Mean × 100%) of each dOCM metric from the pixel-wise values within the segmented ROI for each embryo. The mean CV and standard error of the mean (SEM) across all 34 embryos were calculated.

## 3. Results and discussion

### 3.1. OCM characterization

**Fig. 4.**
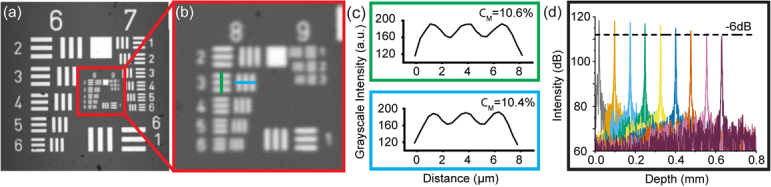
OCM performance characterization: (a) OCM en face projection of a USAF resolution target. (b) Magnified view of the resolution target image. (c) Line profile crosses the bars of Group 8 Element 3 with corresponding Michelson contrast values. (d) Sensitivity fall-off as a function of imaging depth.

Figure 4(a–c) show the OCM en face projections of a USAF resolution target. Group 8, Element 3 was successfully resolved, indicating a lateral resolution of 1.55 µm. The bar visibility was quantified using the Michelson contrast,

(13) $$C_{M}=\frac{I_{max}-I_{min}}{I_{max}+I_{min}},$$

where $I_{\text{max}}$ and $I_{\text{min}}$ denote the averaged peak and valley intensities from the line profiles. Specifically, $I_{max}$ and $I_{min}$ were computed by averaging the peak and valley intensities, respectively, across the line profiles shown in Fig. 4(c). The measured contrast for the smallest resolvable element was approximately 10%, from the grayscale log-scale en face projection, confirming bar visibility. Figure 4(d) shows the SNR fall-off curve. The axial resolution, determined by the averaged full width at half-maximum (FWHM) of the point spread functions (PSFs) across depths, was 2.59 µm in air. Additionally, the measured sensitivity was 110 dB (1 mW at sample, 20 µs integration time), with a 6-dB roll-off depth of approximately 600 µm. Note that the 6-dB roll-off depth is determined by the spectral resolution of the spectrometer and is independent of the objective depth of field. System stability was assessed by tracking the mirror peak position across 100 repeated B-scans. Cross-correlation analysis revealed an axial shift of 0 µm and a maximum lateral shift of 1.18 µm across all frames, remaining within the system lateral resolution of 1.55 µm. The sub-resolution lateral variation is likely due to galvanometer scanner jitter. These results confirm that system-induced motion is negligible over the acquisition window used for embryo imaging.

### 3.2. Correlation between dOCM motility metrics and cell count

**Fig. 5.**
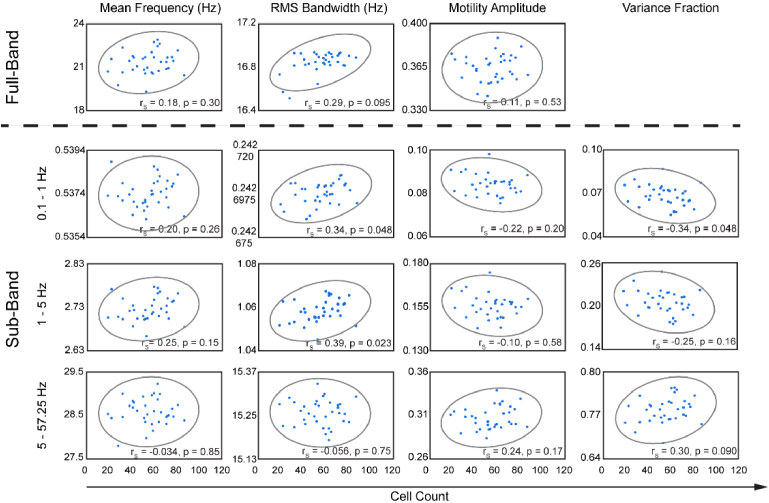
Spearman correlation analysis of dOCM spectral metrics against total cell number (n = 34). Scatter plots illustrating the relationship between embryo cell count (x-axis) and dOCM metrics (y-axis) derived from the full-band (top row) and three frequency sub-bands: low (0.1–1 Hz), mid (1–5 Hz), and high (5–57.25 Hz). No correlations survived Benjamini-Hochberg False Discovery Rate (BH-FDR) correction for multiple comparisons (FDR < 0.05).

The study cohort exhibited a broad distribution of total cell counts, ranging from 19 to 87 cells, which provided a large dynamic range for evaluating associations between dOCM metrics and blastocyst cell count. We evaluated the relationship between the extracted dOCM spectral metrics and total cell number using Spearman’s rank correlation analysis (Fig. 5).

**Fig. 6.**
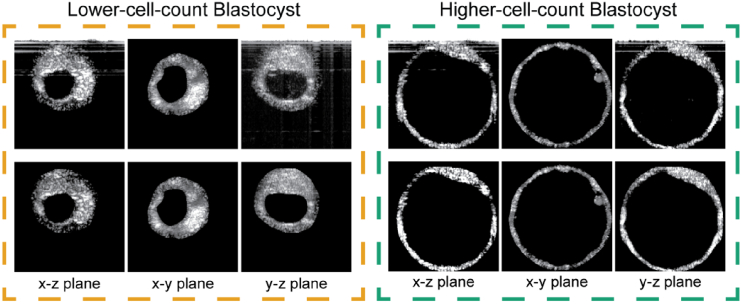
Illustrative structural OCT intensity images of a lower-cell-count blastocyst (left) and a higher-cell-count blastocyst (right) shown in three orthogonal cross-sectional planes (x-z, x-y, and y-z). The top row shows the raw OCT intensity images prior to segmentation, and the bottom row shows the corresponding images after manual segmentation and masking. Each panel corresponds to a 151 × 151 µm field of view.

In the full-frequency band analysis (Fig. 5, top row), positive correlations were observed between cell number and mean frequency, RMS bandwidth, and motility amplitude; however, no statistically significant correlations were observed between cell number and mean frequency (r_s_ = 0.18, p = 0.30), RMS bandwidth (r_s_ = 0.29, p = 0.095), or motility amplitude (r_s_ = 0.11, p = 0.53). These findings indicate that aggregate spectral metrics do not vary systematically with blastocyst quality and suggest that full-band analysis may obscure frequency-dependent changes.

In contrast, band-resolved analysis revealed significant frequency-specific trends. In the low-frequency band (0.1–1 Hz), RMS bandwidth (r_s_ = 0.34, p = 0.048) and variance fraction (r_s_ = −0.34, p = 0.048) exhibited nominally significant correlations with cell number prior to correction, as did mid-frequency RMS bandwidth (r_s_ = 0.39, p = 0.023). After applying BH-FDR correction for multiple comparisons, no correlations in the sub-band analysis survived correction, suggesting that the monotonic relationships between individual dOCM metrics and cell count are modest. No statistically significant correlations were observed for the remaining metrics across the sub-bands. The increase in RMS bandwidth in high-cell-count embryos within both the low- and mid-frequency bands indicates that motions become less spectrally concentrated as cell number increases. These findings suggest that more comprehensive cellular motions were involved in high-cell-count embryos. Concurrently, the reversing trend in variance fraction from decreasing for low frequencies to increasing for high frequencies, though not statistically significant, suggests a redistribution of motion contributions toward faster, small-scale subcellular dynamics with increasing cell number**.**

### 3.3. Band-resolved dOCM metrics distinguish blastocyst quality

To evaluate whether band-resolved dOCM metrics distinguish blastocyst quality using cell count as a proxy, we examined qualitative dOCM maps of embryos to identify visual differences and then performed cohort-level statistical analysis to quantify group differences.

**Fig. 7.**
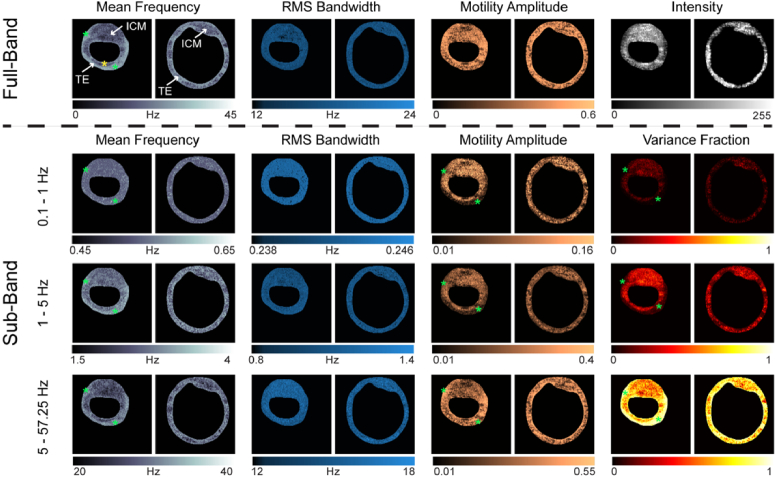
2D maps of mean frequency, RMS bandwidth, motility amplitude, intensity and variance fraction are shown for representative lower-cell-count (left in each pair) and higher-cell-count (right in each pair) blastocysts. Central B-scans were selected to include both the inner cell mass (ICM) and trophectoderm (TE) within the field of view. The ICM and TE are labeled in the full-band mean frequency panels. The top row displays metrics computed across the full frequency spectrum, along with the corresponding structural OCT intensity map to provide morphological context. Subsequent rows present band-resolved analyses for the low- (0.1–1 Hz), mid- (1–5 Hz), and high-frequency (5–57.25 Hz) sub-bands. Mean frequency, RMS bandwidth, and motility amplitude maps illustrate the spatial distribution of localized intracellular dynamics within each frequency range. The variance fraction maps (right column) depict the proportional contribution of each sub-band to the total fluctuation energy at each spatial location. All maps are shown after manual segmentation. The yellow star marks the trophectoderm (TE) region, and the green stars indicate the fluid-filled gap between the TE and the zona pellucida in the lower-cell-count embryo. Each panel corresponds to a 151 × 151 µm field of view.

For visualization, we show two illustrative blastocysts: one from the lower-cell-count group, which correspondingly had a lower Gardner expansion grade (grade 1), and one from the higher-cell-count group, which corresponded with a higher expansion grade (grade 4). All blastocysts were terminated at approximately the same time point; therefore, expansion grade is interpreted here as a measure of quality rather than developmental progression or stage. Figure 6 shows illustrative structural OCT intensity images of both blastocysts in three orthogonal cross-sectional planes (x-z, x-y, and y-z), both before and after manual segmentation, demonstrating sufficient signal throughout the cellular region to support segmentation and dOCM metric extraction. For both samples, we rendered 2D cross-sectional maps of the extracted dOCM metrics (Fig. 7). Central B-scans were selected to include both the ICM and TE within the 151 × 151 µm field of view. The ICM and the TE are labeled in the mean frequency map in Fig. 7. The maps display localized mean frequency, RMS bandwidth, motility amplitude, and variance fraction across the full spectrum and the three defined sub-bands, along with the structural OCT intensity map in the full-band row to facilitate direct comparison between dynamic contrast and underlying morphology.

Both full- and sub-band maps show qualitatively similar intensity between low- and high-cell-count embryos across mean frequency, RMS bandwidth, and motility amplitude. This observation suggests that a high-cell-count embryo does not simply exhibit increased motion magnitude compared to a low-cell-count embryo.

In the low-cell-count embryo, a brighter peripheral region (marked by the green star in Fig. 7) is evident in the mean frequency maps of the full- and high-frequency bands relative to the adjacent inner ring (marked by the yellow star in the full-band mean frequency panel in Fig. 7). This feature is not apparent in the low- and mid-frequency band (0.1–1 Hz). At the blastocyst stage, the zona pellucida (ZP) thins to a thickness approaching the optical resolution limit and exhibits inherently weak backscattering. As such, the ZP itself is unlikely to be directly visualized in these maps. The observed brighter ring in mean frequency, therefore, likely corresponds to the fluid-filled gap between the TE and the ZP, and the dimmer inner ring likely corresponds to TE. Notably, this same peripheral region appears relatively dimmer in motility amplitude and variance fraction maps within the low- and mid-frequency bands, but brighter in the high-frequency band. This band-dependent contrast suggests that the acellular fluid-filled gap is dominated by rapid, diffusion-like fluctuations rather than slower, structure-associated dynamics. Such behavior is consistent with Brownian motion, which is expected to dominate in regions lacking dense cellular structure. This peripheral signature was not observed in the high-cell-count embryo, consistent with structural remodeling and loss of the fluid-filled gap in an advanced embryo. Visual differences were observed in the low- and high- frequency band variance fraction maps. Apart from the fluid-filled gap, the ICM and TE in the low-cell-count embryo exhibited a higher variance fraction in the low-frequency band and a lower variance fraction in the high-frequency band than in high-cell-count embryos. This redistribution of variance suggests a distinct dynamic signature in high-cell-count embryos, with less variance in larger-scale, slower motions and more variance in rapid intracellular dynamics.

**Fig. 8.**
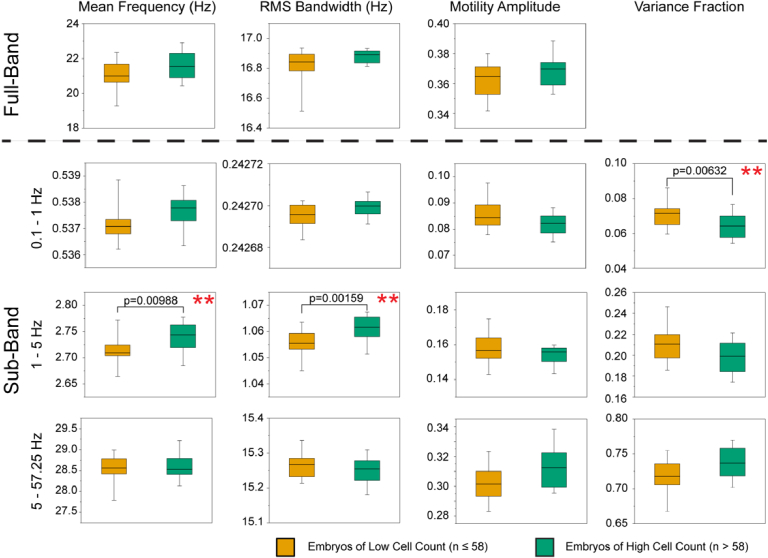
Comparison of band-resolved dOCM motility metrics between lower- and higher-cell-count blastocysts**.** Box plots illustrate the distribution of Mean Frequency, RMS Bandwidth, Motility Amplitude, and Variance Fraction derived from the full frequency spectrum (top row) and three distinct sub-bands: 0.1–1 Hz, 1–5 Hz, and 5–57.25 Hz. The cohort was stratified into two independent groups based on the median cell count: low-cell-count blastocysts (n ≤ 58, orange) and high-cell-count blastocysts (n > 58, green). Group differences were assessed using the two-tailed Mann-Whitney U test, with Benjamini-Hochberg False Discovery Rate (BH-FDR) correction applied across all 15 comparisons. Raw p-values are shown for statistically significant comparisons surviving BH-FDR correction (FDR < 0.05). Significance is indicated by red asterisks: ** p < 0.01 (FDR-corrected).

To determine whether these qualitative trends are consistent across embryos and not driven by slice-specific variability, we next performed cohort-level analysis by stratifying embryos into two groups based on the median cell count. When evaluating the full-frequency fluctuation spectrum, no statistically significant differences were observed between the two cohorts across mean frequency, RMS bandwidth, or motility amplitude (Fig. 8, top row). This result is consistent with the full-band correlation analysis and indicates that high-cell-count blastocysts do not exhibit simple global increases in these dynamic metrics.

Spectral decomposition into discrete frequency sub-bands revealed band-specific differences, highlighting the necessity of isolating distinct intracellular motility regimes. In the mid-frequency band (1–5 Hz), the mean frequency was significantly higher in the high-cell-count group (p = 0.00988). Although mean frequency did not show a statistically significant monotonic relationship with cell number in the corresponding correlation analysis, this result indicates that mean frequency can differentiate the low- and high-cell-count phenotypes at the cohort level.

Furthermore, the mid-frequency band revealed a significant broadening of the RMS bandwidth in the high-cell-count cohort (p = 0.00159), indicating greater spectral diversity of intracellular motion at this timescale. While the absolute motility amplitude (a proxy for motion magnitude) remained similar between the low- and high-cell-count groups across the evaluated bands, the spectral distribution of motion shifted significantly. Variance fraction analysis showed that the high-cell-count group exhibited a significant decrease in the low-frequency (0.1–1 Hz) variance fraction (p = 0.00632), consistent with the qualitative patterns observed in Fig. 7 and the directionality of the correlation analysis. This pattern indicates a redistribution of motion from slower structural dynamics toward faster, small-scale subcellular motion with increasing cell number. After BH-FDR correction for multiple comparisons, no other metrics reached statistical significance.

### 3.4. Intra-embryo spatial heterogeneity of dOCM metrics

To further characterize spatial heterogeneity within individual embryos, we computed the intra-embryo CV of each dOCM metric from pixel-wise values within the segmented ROI and report the mean CV ± SEM across all 34 embryos in Table 2. The metrics fall into two categories with distinct variability profiles. Amplitude-based metrics — motility amplitude and variance fraction — exhibited high intra-embryo CVs across all bands, most notably low-band variance fraction (94.17 ± 0.71%) and low-band motility amplitude (61.83 ± 0.32%), reflecting substantial cell-to-cell variability in dynamic energy levels within individual embryos. This confirms that global ROI averaging collapses meaningful spatial heterogeneity for these metrics, consistent with the weak full-band correlations observed above. In contrast, frequency-based metrics — mean frequency and RMS bandwidth — showed markedly lower intra-embryo CVs, particularly under sub-band decomposition: low-band RMS bandwidth (1.15 ± 0.01%), low-band mean frequency (3.95 ± 0.01%), and mid-band RMS bandwidth (8.52 ± 0.04%). This reduction in intra-embryo CV under sub-band decomposition suggests that isolating specific frequency bands captures consistent and reproducible motility regimes within each embryo, independent of cell-to-cell amplitude variability. These findings support the potential of sub-band frequency metrics as candidate biomarkers that are more robust to spatial heterogeneity than amplitude-based measures.

**Table 2.** Intra-embryo coefficient of variation (CV) of dOCM metrics across all 34 embryos, reported as mean CV% ± Standard Error of the Mean (SEM, n = 34).

Band	Mean frequency (Hz)	RMS bandwidth (Hz)	Motility amplitude	Variance fraction
	Mean CV% ± SEM	Mean CV% ± SEM	Mean CV% ± SEM	Mean CV% ± SEM
**Full band**	26.96 ± 0.36	8.76 ± 0.09	33.23 ± 0.31	
**Low band**	3.95 ± 0.01	1.15 ± 0.01	61.83 ± 0.32	94.17 ± 0.71
**Mid band**	13.55 ± 0.03	8.52 ± 0.04	45.14 ± 0.31	56.75 ± 0.47
**High band**	10.93 ± 0.07	7.36 ± < 0.01	36.49 ± 0.30	23.38 ± 0.37

## 4. Conclusion

This study presents the first demonstration of dOCM for the quantitative quality assessment of murine embryos at the blastocyst stage. In contrast to prior dOCM investigations restricted to the cleavage stage, the present study addresses the blastocyst — a critical developmental stage for clinical IVF transfer. By applying dOCM to murine blastocysts and performing both correlation analysis and cohort stratification, we identified frequency-resolved spectral signatures of intracellular motion that correlate with total cell count and differentiate low- and high-cell-count embryos. Total cell count is an established biomarker associated with blastocyst quality; however, accurate cell counting is typically not accessible in standard IVF workflows because it generally requires staining and fixation. These quantitative, label-free dOCM-derived motility signatures therefore provide a biologically relevant and noninvasive approach for assessing embryo quality.

While full-band dOCM summary metrics — including mean frequency, RMS bandwidth, and motility amplitude — showed no significant relationship with cell number (all p > 0.05), decomposing the signal into sub-bands uncovered frequency-specific trends that were otherwise obscured in the full-spectrum analysis. Collectively, these candidate biomarkers suggest that higher-quality blastocysts may be characterized not by simple increases in motility amplitude, but by a shift toward more complex and spectrally broad intracellular activity, though these findings remain exploratory and require validation in larger cohorts.

Despite these group-level distinctions, several limitations must be considered when interpreting the spectral data. First, the biological attribution of specific frequency bands is currently extrapolated from frequency regimes characterized in 3D tissue models. While this provides a useful theoretical framework, future studies are required to map these spectral bands definitively to specific organelle transport mechanisms within murine embryos. Second, it is important to acknowledge the resolution limits of the dOCM system (∼1.4 µm lateral, ∼2.6 µm axial) relative to the scale of intracellular organelles. Since mitochondria (∼0.5–1 µm) and vesicles (< 500 nm) fall below the optical diffraction limit of the system, the recorded fluctuations do not represent the resolved motion of single distinct organelles. Rather, the dOCM signal represents the interferometric summation of sub-resolution scatterers within each coherence volume, meaning the spectral signatures observed here reflect aggregate ensemble statistics of intracellular transport. Third, the frequency resolution of the spectral analysis is constrained by the temporal sampling scheme at each spatial location. In this study, 100 repeated B-scans were acquired at each B-scan position at a repetition rate of 114.5 Hz, yielding a total sampling window of ∼0.87 s and a frequency resolution of Δf ≈ 1.15 Hz. As a result, sensitivity to slower dynamics, particularly within the low-frequency band (0.1–1 Hz), is limited by the short acquisition window. Increasing the number of repeated B-scans would improve frequency resolution but was constrained in our current system by data volume and computational throughput during acquisition and processing. Each embryo dataset currently requires approximately 26.8 GB of raw storage and more than three hours of total processing time on a standard laboratory workstation (Intel Core i7-9800X, 3.80 GHz, 64 GB RAM, MATLAB 2023a) even with CPU-based parallelization via the MATLAB Parallel Computing Toolbox. Increasing the number of B-scans would proportionally increase both requirements, and GPU-accelerated processing and optimized acquisition protocols represent important directions for future work. Because the same sampling protocol was applied to all embryos, this limitation does not bias the correlation analysis and comparisons between cohorts. Fourth, the reported biomarkers were derived via global ROI averaging to maximize signal robustness for correlation testing. Although the system achieves a lateral resolution of 1.55 µm, individual cell boundaries within the blastocyst cannot be resolved due to the low refractive index contrast between adjacent cells, precluding single-cell level analysis. This global averaging approach may therefore mask spatially heterogeneous signatures, such as distinct metabolic activity differences between the TE and the ICM, as well as cell-to-cell variability in dynamic activity within each blastocyst. As shown in Table 2, amplitude-based metrics exhibited high intra-embryo CVs, confirming that global averaging collapses meaningful spatial heterogeneity for these metrics. Frequency-based metrics showed markedly lower intra-embryo CVs, particularly under sub-band decomposition, suggesting they are more robust to this limitation. Future studies could also explore pixel-wise dynamic scoring and spatially resolved analysis of distinct embryo compartments — such as the ICM and TE separately — to determine whether localized dOCM signatures provide stronger associations with blastocyst quality than global ROI averages. Fifth, although total cell count serves as a robust proxy for blastocyst quality, the direct relationship between these dOCM spectral signatures and implantation potential or live birth outcomes has not yet been established. Future longitudinal studies incorporating embryo transfer models are necessary to validate the predictive value of dOCM for pregnancy outcomes. Sixth, while no visible embryo motion was observed during imaging, subtle sample motion — particularly in the y-direction — cannot be fully excluded and has not been thoroughly studied. Future work incorporating image registration or motion correction strategies would further improve the robustness of the dynamic metrics. Seventh, no sample dispersion correction was applied; while the self-interference configuration eliminates reference-sample arm dispersion mismatch, residual sample dispersion from the embryo and culture media may affect axial resolution. Finally, the phototoxicity of the dOCM imaging protocol has not been explicitly validated. As this is an exploratory study, careful validation of photosafety will be required before clinical translation.

Overall, these results suggest that band-resolved dOCM can provide a quantitative, label-free functional readout of blastocyst quality that is not captured by standard morphological grading alone. By enabling quantitative embryo assessment based on intracellular dynamics, this approach could complement existing grading methods and improve embryo selection in IVF, potentially supporting higher implantation success.

## Data Availability

The data supporting the results of this study are available from the corresponding author upon reasonable request.
